# Mass Spectrometry-Based Metabolomics Investigation on Two Different Seaweeds Under Arsenic Exposure

**DOI:** 10.3390/foods13244055

**Published:** 2024-12-16

**Authors:** Yuan-sheng Guo, Shuo Gong, Si-min Xie, An-zhen Chen, Hong-yu Jin, Jing Liu, Qi Wang, Shuai Kang, Ping Li, Feng Wei, Tian-tian Zuo, Shuang-cheng Ma

**Affiliations:** 1 National Institutes for Food and Drug Control, State Key Laboratory of Drug Regulatory Science, Beijing 100050, China; gysheng2022@163.com (Y.-s.G.); jhyu@nifdc.org.cn (H.-y.J.); liujing_zsm@126.com (J.L.); sansan8251@sina.com (Q.W.); kangshuai@nifdc.org.cn (S.K.); weifeng@nifdc.org.cn (F.W.); 2 School of Pharmacy, China Pharmaceutical University, Nanjing 211198, China; liping2004@126.com; 3 School of Integrative Medicine, Anhui University of Chinese Medicine, Hefei 230012, China; gongshuo0221@163.com; 4 Guangzhou Institute for Drug Control, Key Laboratory for Quality Evaluation of Chinese Patent Medicine, National Medical Products Administration, Guangzhou 510160, China; xiesimingz@163.com; 5 Qingdao Institute for Food and Drug Control, NMPA Key Laboratory for Quality Research and Evaluation of Traditional Marine Chinese Medicine, Qingdao 266073, China; 87666368@163.com; 6 Chinese Pharmacopoeia Commission, Beijing 100061, China

**Keywords:** seaweeds, arsenic, metabolomics, LC-MS, pathways

## Abstract

Arsenic is a common toxic heavy metal contaminant that is widely present in the ocean, and seaweeds have a strong ability to concentrate arsenic, posing a potential risk to human health. This study first analyzed the arsenic content in two different seaweeds and then used an innovative method to categorize the seaweeds into low-arsenic and high-arsenic groups based on their arsenic exposure levels. Finally, a non-targeted metabolomic analysis based on mass spectrometry was conducted on seaweed from different arsenic exposure groups. The results indicated that as the arsenic concentration increased in the seaweeds, linolenic acid, tyrosine, pheophorbide a, riboflavin, and phenylalanine were upregulated, while arachidonic acid, eicosapentaenoic acid (EPA), betaine, and oleamide were downregulated. The following four key metabolic pathways involving unsaturated fatty acids and amino acids were identified: isoquinoline alkaloid biosynthesis, tyrosine metabolism, phenylalanine metabolism, and riboflavin metabolism. The identification of biomarkers and the characterization of key metabolic pathways will aid in the selection and breeding of low-arsenic-accumulating seaweed varieties, providing insights into the metabolic and detoxification mechanisms of arsenic in seaweeds.

## 1. Introduction

Arsenic is a pervasive toxic metalloid found in the environment, exerting varying degrees of toxicity on plants, animals, and humans [1]. It occurs in multiple forms in nature, including inorganic arsenic such as arsenite (As(III)) and arsenate (As(V)), and organic arsenic such as arsenobetaine (AsB), arsenocholine (AsC), monomethylarsonate (MMA), and dimethylarsinate (DMA) [2,3]. Among these, inorganic arsenic is significantly more toxic than organic arsenic [4,5]. Prolonged exposure to arsenic, particularly inorganic forms, can lead to damage to vital organs such as the kidneys, liver, and stomach, as well as skin lesions and various types of cancer [6,7]. Recent studies suggest that long-term, low-dose arsenic exposure may be associated with chronic diseases such as cardiovascular diseases, diabetes, and neurodegenerative disorders [8,9]. According to data from the World Health Organization (WHO), over 45 million people in Bangladesh are exposed to drinking water with high concentrations of arsenic [10]. Long-term consumption of water with elevated arsenic levels can result in a range of health issues, including skin, lung, and bladder cancer [11].

Arsenic can enter the human body through various pathways, primarily including drinking water, food, air, and soil. Once arsenic enters the food chain or drinking water, it poses a threat to both animal and human health [12]. Therefore, studying the presence of arsenic in food, which includes both plant and animal sources, is crucial for preventing and mitigating the toxic effects of arsenic. However, a limited number of specific regulations are in place regarding the arsenic content in seaweed worldwide. For instance, in China, the “Chinese Pharmacopoeia” (2020 edition) records the maximum allowable limits of arsenic in botanical medicines, stipulating that the maximum residue level of arsenic in these medicines should not exceed 5.0 mg/kg [7,13]. Australia and New Zealand have set a threshold for inorganic arsenic content in seaweed at 1 mg/kg, while France has established a maximum limit of 3 mg/kg for inorganic arsenic in seaweed [14]. Research indicates that the arsenic content in seaweed is significantly higher than the standard, particularly in *Sargassum fusiforme* (Harv.) Setch., whose arsenic content far exceeds the maximum allowable limit set by the “Green Food: Algae and Their Products” standard [15,16]. Moreover, the main arsenic compound in *Sargassum fusiforme* (Harv.) Setch. is the more toxic inorganic arsenic, warranting widespread attention [17]. In response to this situation, researchers have proposed several strategies to reduce the accumulation of arsenic in seaweed, including controlling arsenic concentrations in the environment, employing physicochemical methods to reduce arsenic levels in seaweed, utilizing bioremediation techniques to prevent arsenic from entering the food chain, and reducing industrial emissions [18,19]. Some of these methods are time-consuming, costly, and not easily achievable. Additionally, the use of nanomaterials, such as titanium dioxide nanoparticles, to remove arsenic from seaweed requires further investigation [20]. Therefore, understanding the inherent characteristics of seaweed with low arsenic accumulation is one of the most effective methods to reduce the health risks of arsenic to humans by selecting and cultivating seaweed varieties that accumulate less arsenic.

Studying the arsenic exposure levels of different seaweeds in the environment and elucidating the potential biomarkers and metabolic pathways under various arsenic exposure conditions can help us understand the characteristics of seaweeds with low arsenic accumulation, providing valuable strategies for selecting and breeding seaweed varieties that accumulate less arsenic. In recent years, high-resolution non-targeted metabolomics has become one of the powerful tools for the chemical component analysis of complex multi-component systems such as food and traditional Chinese medicine [21,22]. It not only allows for the acquisition of the metabolic product composition and primary metabolic pathway information from complex systems but also enables the differential analysis of target components in conjunction with chemometric methods. Huang Hui-Yu and colleagues identified 59 upregulated and 52 downregulated metabolites through non-targeted metabolomics, further investigating the mechanism of Citrus grandis leaves in response to copper toxicity in combination with transcriptomics [23]. Mette Jensen and colleagues, through their research on the relationship between heavy metal exposure and metabolic changes in migratory birds (specifically the short-billed goose), found that lipid signaling was negatively correlated with the chromium (Cr) concentration and positively correlated with mercury (Hg) exposure [24].

**Figure 1:**
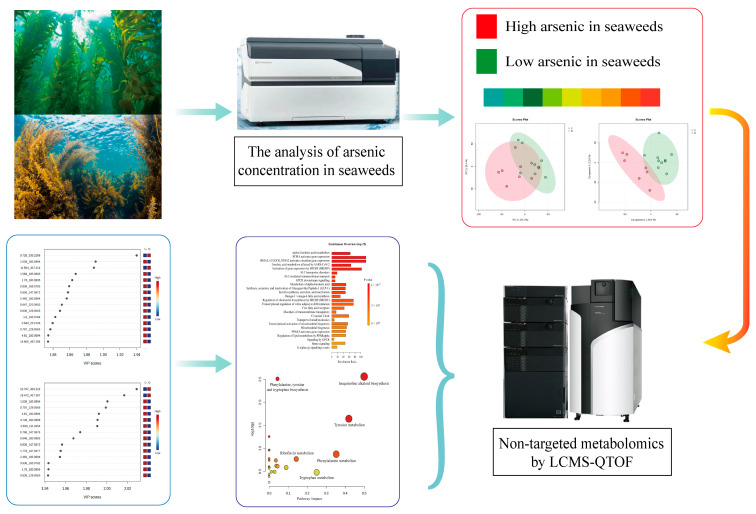
Schematic diagram of mass spectrometry-based metabolomics investigation on two different seaweeds under arsenic exposure.

Therefore, this study aims to explore the differences in the chemical composition of seaweed under various arsenic exposure conditions using high-resolution mass spectrometry. The goal is to uncover the intrinsic chemical characteristics of seaweed, identify potential biomarkers and metabolic pathways, and to provide scientific evidence for selecting and cultivating seaweed varieties with low arsenic accumulation (Figure 1). Additionally, this research seeks to offer insights into the metabolic and detoxification mechanisms of arsenic in seaweed.

## 2. Materials and Methods

### 2.1. Chemicals and Materials

Chemical reagents used for the metabolomic analysis included acetonitrile (LC-MS grade, Merck KGaA, Darmstadt, Germany), formic acid (LC-MS grade, Macklin Biochemical Co., Ltd., Shanghai, China), and methanol (LC-MS grade, Sinopharm Chemical Reagent Co., Ltd., Shanghai, China). Ultrapure water for all experiments was provided by a Mill-Q system (Millipore, Chicago, IL, USA). The rest of the chemicals (analytical grade) were obtained from Sinopharm Chemical Reagent Beijing (Beijing, China). A single-element calibration standard solution (As) was obtained from Sinopharm Chemical Reagent Beijing (Beijing, China). Tuning solutions containing Li, Y, Ce, Tl, and Co (Part# 5185-5959) and internal standard solutions containing Li, Ge, Rh, In, Tb, Lu, and Bi (Part# 5188-6525) were purchased from Agilent (Agilent Technologies, Folsom, CA, USA). Supra-pure trace metal-grade concentrated nitric acid (HNO_3_, 65.0%) was purchased from Merck (Merck, Munchen, Germany).

### 2.2. Arsenic Concentration Determination in Seaweed Samples

#### 2.2.1. Sample Preparation

**Figure 2:**
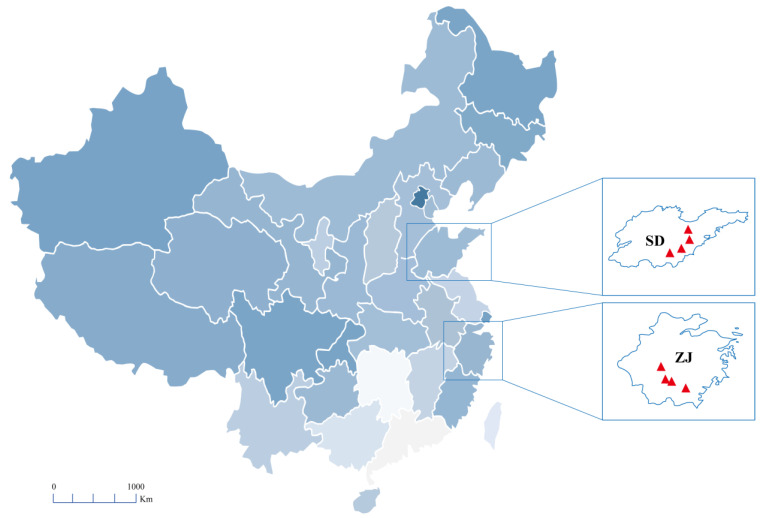
Geographic sites of seaweed samples from different regions in China (SD, Shandong; ZJ, Zhejiang; Specific regions where the samples were collected, marked by the red triangles.)

Seventeen batches of seaweed were collected from TCM markets and origins in the provinces of Shandong and Zhejiang in China in 2022 (Table 1 and Figure 2). After sample collection, the samples were washed, dried, rapidly frozen, and stored at −80 °C. All samples of seaweed were authenticated by Dr. Shuai Kang.

For the determination of arsenic in seaweeds, the sample preparation method followed the guidelines outlined in the Chinese Pharmacopoeia for element analysis. First, PTFE digestion tubes were soaked in a 10% nitric acid solution for 24 h, followed by rinsing with ultrapure water to minimize any instrument interference. Next, 0.3 g of the powdered sample was precisely weighed and placed into a microwave digestion vessel. Then, 7 mL of nitric acid was added to the vessel. The digestion system was then assembled according to the standard procedure and placed in a microwave digestion unit. The gradient temperature program followed, with the temperature increasing from ambiance to 120 °C at the rate of 16 °C/min, followed by a plateau for 3 min at 120 °C; then, the temperature was increased from 120 °C to 150 °C at a rate of 4 °C/min, followed by a plateau for 3 min at 150 °C. Finally, a temperature increase from 150 °C to 190 °C at a rate of 7 °C/min was implemented, followed by a final plateau for 30 min at 190 °C. The power used was 1600 W. After digestion, the sample was cooled to room temperature, excess acid was removed, and the sample was diluted with ultrapure water to a final volume of 50 mL for element analysis.

#### 2.2.2. Calibration Procedure

External calibration curves were followed for the quantitative analysis of samples. For ICP-MS, standard solutions were prepared in 5% (*w*/*w*) HNO_3_ by diluting a multi-element standard solution containing arsenic (As) elementals at concentrations of 100.00, 500.00, 1000.00, 5000.00, 10,000.00, 50,000.00, and 100,000.00 μg/L and the calibration curves were plotted from the limits of detection of the corresponding elements. The internal standards containing Ge (500 ng/mL) were determined concurrently with multiple elements to ensure stability and accuracy for the analysis.

#### 2.2.3. ICP-MS Analysis

The As content was determined using a SHIMADZU 2030 inductively coupled plasma mass spectrometry device (ICP-MS, SHIMADZU., Shanghai, China). The instrumental parameters were set as follows: the high-frequency plasma power was 1250 W with a detector voltage of -1850 V, and high-purity argon (Ar, 99.999%) was used as the carrier gas. The plasma, atomization, and collision gas flow rates were 17 L/min, 0.92 mL/min, and 4.5 mL/min, respectively. The peak hopping mode was selected as the acquisition method with three sampling repeats.

#### 2.2.4. Method Assurance

The ability of the method to determine the content of arsenic in seaweed samples was evaluated using the limits of detection (LODs), limits of quantification (LOQs), spiking recovery, and precision, as shown in Table S1. These results demonstrated that the method enables accurate measurement of the levels of these elements in the seaweed samples.

### 2.3. Metabolite Extraction

For the untargeted metabolomic analysis, the samples were first ground into a fine powder and passed through a 3-mesh sieve. Then, 2.0 g of the powder was precisely weighed and placed into a 50 mL conical flask. To this, 20 mL of 70% methanol was added, and the mixture was vortexed for 10 min, followed by ultrasonic extraction for 45 min. The volume was then adjusted to compensate for any loss during the extraction, and the sample was shaken thoroughly. Afterward, the mixture was centrifuged at 8000 rpm for 10 min, and the supernatant was collected. The supernatant was filtered through a 0.22 µm micropore filter. In addition, quality control (QC) samples were prepared by combining equal volumes of all the seaweed samples.

### 2.4. Non-Targeted Metabolomics by LCMS-QTOF

A SHIMADZU UPLC system (SHIMADZU., Ltd., Shanghai, China) coupled with a quadrupole time-of-flight mass spectrometer (QTOF-MS, SHIMADZU., Ltd., Shanghai, China) was used for analysis. An ACQUITY UPLC HSS C18 column (100 mm × 2.1 mm, 1.8 μm, Waters, Ireland) was applied. The mobile phase consisted of two solvents, including 0.1% formic acid in purified water for phase A and 0.1% formic acid in acetonitrile (ACN) for phase B. The gradient elution procedure was set as follows: 0–6 min, 5–35% B; 6–9 min, 35–65% B; 6–19 min, 65–80% B; 19–28 min, 100% B; and 28–31 min, 100–5% B. The column temperature was kept at 40 °C. The injection volume was 2 μL, and the flow rate was set at 0.4 mL/min.

The mass spectrometer conditions involved using an electrospray ionization source (ESI) as the ion source, operating in both positive and negative modes. The ion source temperature was maintained at 250 °C. The interface voltage and corona needle voltage were set to 4.0 kV and 4.5 kV, respectively. The flow rates of the atomized and heating gasses were 5.0 L/min and 10.0 L/min, respectively, with an interface temperature of 300 °C. All MS data were collected over the *m*/*z* range of 100 to 1200. The NaI (*m*/*z* 172.8834, *m*/*z* 322.7777, *m*/*z* 472.6719, *m*/*z* 622.5662, *m*/*z* 922.3547) was used for real-time calibration of the mass spectra. Additionally, to assess the stability of the UPLC-Q-TOF/MS system and ensure the accuracy of the MS data, QC samples from each algae origin were injected at designated intervals before, during, and after the injection process.

### 2.5. Preprocessing of MS Data

Raw MS data, including peak alignment, peak picking, peak filtering, and data normalization, were processed using MS-DIAL software (version 5.1.2, USA). The parameter settings were as follows: In the data collection section, we used an accurate mass tolerance of 0.010 Da for MS1 and 0.025 Da for MS2, a retention time from 1 min to 33 min, a mass range from 100 Da to 1200 Da for MS1, and a mass range from 50 Da to 1200 Da for MS/MS. In the peak detection section, the minimum peak height was 3000 amplitude, the mass slice width was 0.1 Da, the linear weighted moving average was selected using the smoothing method, the smoothing level was 3 scans, and the minimum peak width was 5 scans. In the spectrum deconvolution, a sigma window value of 0.5 was used, and the MS/MS abundance was cut off from 3000 amplitudes. In the identification section, MSMS_Public_EXP_Pos_VS17 was used as a database, and the accurate mass tolerance was 0.01 Da for MS1 and 0.025 Da for MS2. All preprocessed data were analyzed using principal component analysis (PCA) and partial least squares discriminant analysis (PLS-DA) to identify differential metabolites among different seaweeds.

### 2.6. Statistical Analysis

Data pretreatments, including peak filtering, alignment, identification, and normalization, were conducted using MS-DIAL, the MS-DIAL Database; LabSolutions Insight Explore were used for structure identification. PCA, PLS-DA, orthogonal partial least squares discriminant analysis (OPLS-DA) models and one-way analysis of variance (ANOVA) of the elements and metabolites were used to visualize the distribution trends of algae samples of different origins and were performed using Chempattern 2017 pro software (Chemmind, China) and the MetaboAnylyst (version 6.0, USA). Statistical analyses were performed using Microsoft Excel 2016 (Microsoft Co., USA). The Variable Importance in Projection (VIP) and Fold Change (FC) values were calculated. Among these metabolites, VIP > 1.0 and FC >2 were used as the selection criteria.

## 3. Results and Discussion

### 3.1. Arsenic Concentration

**Figure 3:**
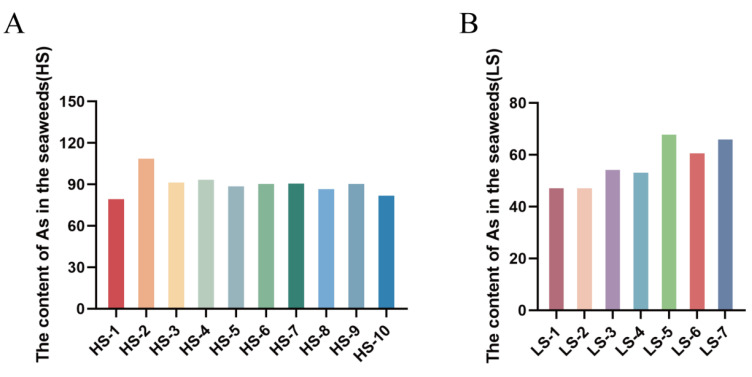
The concentration of As in high-arsenic seaweed samples (**A**) and low-arsenic seaweed samples (**B**). (HS, High-arsenic seaweed samples; LS: Low-arsenic seaweed samples).

In this study, we first measured the arsenic content in the collected seaweed samples using ICP-MS, with the results being presented in Figure 3. The study revealed significant variation in arsenic levels among different species of *Sargassum fusiforme (Harv.) Stech*. Based on these differences and using a threshold of 70 mg/kg for arsenic content, the seaweed samples were classified into two groups, high-arsenic seaweed and low-arsenic seaweed. Previous studies have shown that the primary form of arsenic in *Sargassum fusiforme (Harv.) Stech*. is inorganic arsenic, which suggests that higher arsenic contents in seaweed may correlate with increased arsenic toxicity. For example, a study on arsenic speciation in seaweeds by Huang et al. revealed that the total arsenic concentration in brown algae (0.80–250 mg/kg) was significantly higher than those in red algae (0.13–50 mg/kg) and green algae (0.10–30 mg/kg). Notably, within the brown algae group, species of the Sargassum genus contained higher concentrations of inorganic arsenic (15.1–83.7 mg/kg), which is particularly concerning [13,25,26]. In our study, in the low-arsenic seaweed group, the average arsenic content was 56.78 mg/kg, while in the high-arsenic seaweed group, the average arsenic content was 91.56 mg/kg. These results indicate significant variations in arsenic concentrations among different species of *Sargassum fusiforme (Harv.) Stech.*, which is consistent with previous reports [13].

### 3.2. Metabolomic Analysis Based on UPLC-Q-TOF/MS

#### 3.2.1. Metabolic Profiles

Building on previous research, we developed an untargeted metabolomic strategy based on UPLC-Q-TOF/MS to obtain comprehensive metabolite information. The total ion chromatograms (TICs) of representative seaweed samples are shown in Figures S1 and S2. The results indicated that a total of 9752 features were obtained in positive ion mode and 6289 features in negative ion mode on the Q-TOF/MS, demonstrating that the established method is effective for the comprehensive analysis of metabolites in seaweed. Based on the prior classification of low-arsenic and high-arsenic seaweed groups and given the complexity and similarity of the metabolites in seaweed, we plan to apply chemometrics to further explore the differences in metabolites across different seaweed species and assess how varying arsenic content levels affect the seaweed metabolome.

#### 3.2.2. PCA and PLS-DA Models

**Figure 4:**
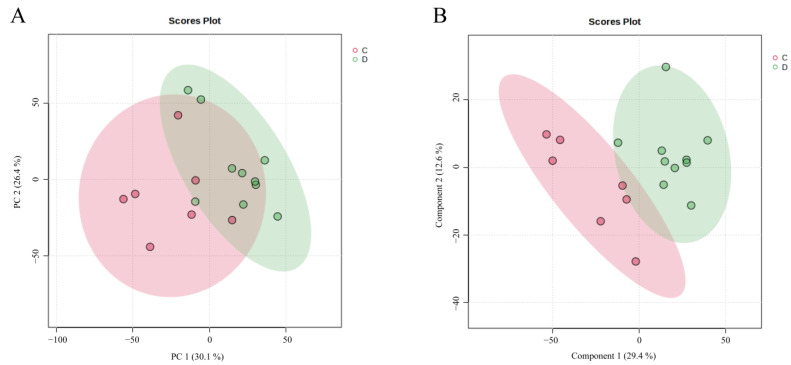
Score plot of PCA model (**A**); score plot of PLS-DA Model (**B**).

In recent years, chemometric techniques, including PCA and PLS-DA, have been widely used for data analysis in untargeted metabolomics. In this study, the raw mass spectrometry data were initially processed using MS-DIAL, which included peak extraction, alignment, and other steps, resulting in a data matrix of 42 × 9754. The processed data were then imported into the MetaboAnalyst platform, where a principal component analysis (PCA) model was constructed for seaweed samples of different origins. A total of five principal components were extracted, with a cumulative explained variance (R²X) of 0.797, indicating that the model effectively explained the data. As shown in Figure 4A, seaweed samples from the high-arsenic and low-arsenic exposure groups clustered independently, achieving good separation. This suggests significant differences in the metabolites of seaweed between the two arsenic exposure groups.

Building on this, a supervised PLS-DA model was applied to achieve a more robust classification. In the PLS-DA analysis, the cumulative explained variance (R²X) of the first five principal components was 0.751, indicating that the preprocessed data effectively differentiated seaweed samples from the different arsenic exposure groups (Figure 4B). This result was consistent with the classification outcomes of the PCA model. The major biomarkers in seaweed from the two different arsenic exposure groups are summarized in Table S2.

### 3.3. Metabolic Pathway

**Figure 5:**
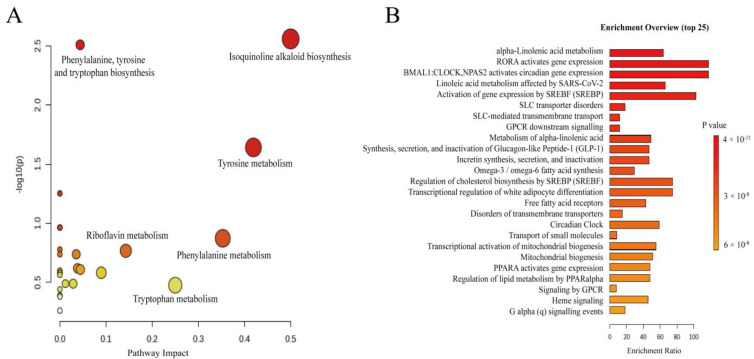
Metabolic pathways (**A**) and enrichment pathways (**B**) from the analysis of seaweed samples from different arsenic exposure groups. (Size of bubbles represents influence of pathway).

After performing chemometric analysis, a metabolic pathway analysis was conducted on the identified differential metabolites. The primary enriched metabolic pathways in the seaweed samples from different arsenic exposure groups include the biosynthesis of unsaturated fatty acids; biosynthesis of isoquinoline alkaloids; biosynthesis of phenylalanine, tyrosine, and tryptophan; α-linolenic acid metabolism; tyrosine metabolism; linoleic acid metabolism; glycine, serine, and threonine metabolism; arachidonic acid metabolism; phenylalanine metabolism; riboflavin metabolism; fatty acid biosynthesis; citric acid cycle; and starch and sucrose metabolism. These pathways were found to be significantly impacted. The metabolic pathways of seaweed from different arsenic exposure groups are illustrated in Figure 5.

### 3.4. Targeted Profiles

**Figure 6:**
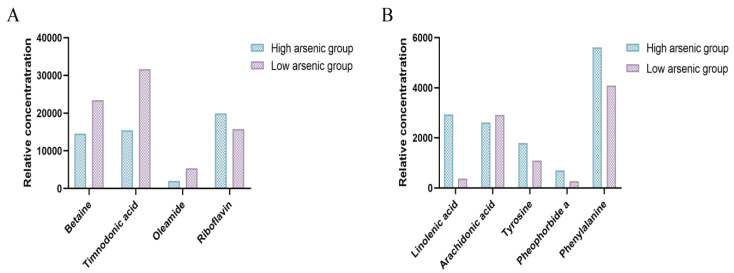
Relative intensities of metabolites in seaweeds under different arsenic exposures. ((**A**): the relative intensities of betaine, timnodonic acid, oleamide, and riboflavin; (**B**): the relative intensities of linolenic acid, arachidonic acid, tyrosine, pheophorbide a, and phenylalanine).

The mass spectrometry data were qualitatively analyzed using public databases such as HMDB and PubChem, as well as relevant studies. By comparing the data from both the full-scan and MS/MS spectra, along with retention times, target metabolites were identified. In both positive and negative ion modes, a total of 72 compounds were identified, including unsaturated fatty acids, lipids, amino acid derivatives, and esters. To select characteristic metabolites for different seaweed groups, the filtering criteria were set as VIP > 1.5 and FC > 2, resulting in the identification of the following nine differential metabolites: linolenic acid, arachidonic acid, betaine, tyrosine, phenylalanine, timnodonic acid, oleamide, pheophorbide a, and riboflavin. The relative intensities of these compounds are shown in Figure 6. The results indicate that with increasing arsenic concentrations, the expressions of linolenic acid, tyrosine, pheophorbide a, phenylalanine, and riboflavin were upregulated, while the expressions of unsaturated fatty acids, such as timnodonic acid, arachidonic acid, and oleamide, as well as betaine, were downregulated.

Among these, linolenic acid, an important unsaturated fatty acid, is a component of human cells and plays a role in fat metabolism. It can be converted into EPA (eicosapentaenoic acid) and DHA (docosahexaenoic acid), both of which have important immune and anti-inflammatory functions [27,28]. As the arsenic levels in seaweed increase, the concentration of linolenic acid also rises, suggesting that linolenic acid metabolism is more pronounced in seaweed with higher levels of arsenic accumulation. Tyrosine is involved in the synthesis of various metabolic products in plants and showed a positive correlation with the arsenic content in seaweed, indicating that as the arsenic concentration increases, tyrosine-related metabolic pathways also intensify. Pheophorbide a, an intermediate product of chlorophyll degradation, has been shown to exhibit significant antiviral activity against certain viruses, such as hepatitis C virus (HCV) and SARS-CoV-2 [29]. Phenylalanine, which is metabolized through several enzymes in the body, can be converted into tyrosine, and it is involved in the synthesis of several important biomolecules [30,31]. As a precursor to the synthesis of various vital compounds, its metabolic reactions also increase with higher arsenic levels, which is consistent with the findings reported in the literature [32,33]. At the same time, algae may increase the metabolic products of tyrosine and phenylalanine, which have antioxidant properties that can help mitigate the oxidative damage induced by arsenic [34]. Riboflavin is a component of two important coenzymes—flavin mononucleotide (FMN) and flavin adenine dinucleotide (FAD)—which are involved in energy metabolism in the body, including fatty acid metabolism and the citric acid cycle [35]. They also play a role in the metabolism of fats and carbohydrates, contributing to overall health maintenance [36]. As arsenic concentrations increase, riboflavin becomes activated to help counteract arsenic-induced damage. Timnodonic acid, a type of ω-3 polyunsaturated fatty acid, is an essential nutrient in humans and is involved in the biosynthesis of ω-3 fatty acids, the citric acid cycle, and biogenic amine metabolism [37]. It significantly reduces levels of metabolites such as creatinine. However, the timnodonic acid expression in algae decreased with higher arsenic levels, potentially due to arsenic stress [38,39]. Arachidonic acid participates in several metabolic pathways, including the cyclooxygenase (COX), lipoxygenase (LOX), and cytochrome P450 (CYP450) pathways. These pathways lead to the production of various bioactive mediators that play crucial roles in both physiological and pathological conditions, including inflammation and vascular regulation [40,41]. Additionally, as the arsenic concentration increases, arsenic may reduce the synthesis of arachidonic acid by inhibiting enzymes that are involved in fatty acid synthesis, such as fatty acid synthase (FAS) [42]. Oleamide, an endogenous fatty acid amide, has been shown to be downregulated in algae under arsenic stress [43]. Due to its antioxidant properties, the decrease in oleamide levels with increasing arsenic concentrations indicates a decline in the seaweed’s ability to tolerate arsenic. Betaine is an important osmoregulatory compound that is produced by plants under stress, and it plays a vital role in regulating the cell osmotic pressure and maintaining the integrity of cell membranes, enzymes, and proteins. Betaine helps plants resist heavy metal stress, and its levels are generally high in seaweeds [44,45]. However, in the presence of arsenic, betaine may bind with arsenic to form arsenobetaine, potentially converting toxic inorganic arsenic into non-toxic organic arsenic, thus serving as a detoxification mechanism.

Research has shown that seaweed, especially *Sargassum fusiforme (Harv.) Stech*., has a unique ability to accumulate arsenic [46]. Moreover, the arsenic contents in different seaweed species vary widely. Therefore, it is crucial to analyze seaweeds that have been exposed to different levels of arsenic, using non-targeted metabolomics techniques, to identify the underlying causes of these variations. This will provide valuable insights for selecting and cultivating seaweed varieties with low arsenic contents, offering useful guidance for public health and safety. Research shows that the most significant metabolic pathways influencing the classification of seaweed in different arsenic exposure groups are those related to unsaturated fatty acids and amino acids. As the arsenic concentration increases, the expression of linolenic acid is upregulated, activating the biosynthesis and metabolism of alpha-linolenic acid. This metabolic process may produce a series of important bioactive compounds that play a crucial role in helping seaweed resist arsenic stress. Meanwhile, the expressions of arachidonic acid and timnodonic acid (EPA) are downregulated. Arachidonic acid metabolism appears to be influenced by arsenic stress, with its metabolic rate accelerating and generating more immune-active substances through various enzyme systems (the COX, LOX, and CYP450 pathways) to counter arsenic stress, which in turn lowers its own concentration [47]. Similarly, EPA is also involved in these metabolic pathways, leading to the downregulation of both arachidonic acid and EPA expressions.

Amino acid-related metabolic pathways also play a crucial role in seaweed exposed to different levels of arsenic. First, tyrosine metabolism involves multiple pathways, including the synthesis of tocopherols, plastoquinone, ubiquinone, and others, as well as serving as a precursor for various secondary metabolites [48]. Similarly, phenylalanine metabolism also participates in the synthesis of several metabolites [49]. Both biosynthetic pathways begin with the shikimate pathway, which produces the common intermediate chorismate, which is then converted into phenylalanine, tyrosine, and tryptophan through the actions of different enzymes [50]. Additionally, both pathways influence the isoquinoline alkaloid biosynthesis, a class of alkaloids that are abundant in the plant kingdom with significant pharmacological and bioactive properties, such as anticancer, antioxidant, and anti-inflammatory activities [51]. As a result, under the influence of these various metabolic pathways, the expression of tyrosine metabolism and phenylalanine metabolism increases with higher arsenic concentrations, enabling seaweed to better cope with arsenic-induced stress. Furthermore, aside from these key metabolic pathways, riboflavin, a key differential metabolite, plays an important role in riboflavin metabolism. As an electron carrier, riboflavin participates in energy production and various metabolic processes [36]. Therefore, in high-arsenic-exposure seaweed, the expression of riboflavin also increases, helping generate more energy to support the plant in effectively responding to environmental stress.

## 4. Conclusions

To date, effective solutions to the issue of excessive arsenic levels in seaweed remain scarce. The complexity and diversity of chemical components in seaweed increase the difficulty of identification, posing challenges to research on seaweed. Previous studies have often focused solely on the chemical composition of seaweed, using relatively outdated research methods. This study used an innovative method of taking arsenic exposure as the starting point and employing high-resolution metabolomics technology to analyze seaweed from different arsenic exposure groups. The results indicate that the metabolic pathways of unsaturated fatty acids and amino acids are key pathways that respond to arsenic. With increasing arsenic concentrations, the metabolic pathways related to arachidonic acid and timnodonic acid are activated, accelerating the metabolic process and downregulating their expression. The expression of linolenic acid is upregulated, which may enhance the seaweed’s tolerance to arsenic toxicity. Amino acids such as tyrosine metabolism and phenylalanine show upregulated expression to resist arsenic stress. In the high-arsenic seaweed group, the expression of riboflavin increases, possibly because the plants’ metabolic reactions and energy requirements increase under adverse conditions. Therefore, when selecting and cultivating low-arsenic seaweed varieties, compounds that play a key role in the breeding process include amino acid derivatives such as betaine, unsaturated fatty acids such as arachidonic acid and timnodonic acid, and amines such as oleamide. These compounds are upregulated in low-arsenic seaweeds, but their expression may decrease as the arsenic concentration increases. In contrast, high-arsenic seaweeds activate metabolic pathways such as the tyrosine, phenylalanine, and riboflavin metabolic pathways, which may be linked to the metabolism of α-linolenic acid and the production of jasmonic acid. Amino acids such as phenylalanine and tyrosine, as well as riboflavin, linolenic acid, and pheophorbide a, show increased expressions with rising arsenic levels, which may enhance the seaweed’s tolerance to arsenic stress. Through our research, the internal chemical component differences in seaweed samples from different arsenic exposure groups and various biomarkers have been identified, and key metabolic pathways have been determined, providing a reference for further research on arsenic metabolism and detoxification mechanisms in seaweed and offering theoretical guidance for the selection and cultivation of safe, non-toxic, low-arsenic seaweed varieties.

## Figures and Tables

**Table 1:** Sample collection information of the present study.

No.	Batch No.	Location	Source
1	HS-1	Zhejiang	Origin
2	HS-2	Shandong	TCM market
3	HS-3	Shandong	TCM market
4	HS-4	Zhejiang	TCM market
5	HS-5	Shandong	TCM market
6	HS-6	Zhejiang	TCM market
7	HS-7	Zhejiang	TCM market
8	HS-8	Shandong	TCM market
9	HS-9	Shandong	TCM market
10	HS-10	Shandong	TCM market
11	LS-1	Shandong	Origin
12	LS-2	Zhejiang	Origin
13	LS-3	Zhejiang	Origin
14	LS-4	Zhejiang	Origin
15	LS-5	Zhejiang	Origin
16	LS-6	Shandong	Origin
17	LS-7	Zhejiang	TCM market

## Data Availability

The original contributions presented in this study are included in the article and Supplementary Material. Further inquiries can be directed to the corresponding authors.

## Supplementary Materials

The following supporting information can be downloaded at: https://www.mdpi.com/article/10.3390/foods13244055/s1, Figure S1: The total ion chromatograms (TIC) of representative seaweed samples (QC) based on UPLC-Q-TOF/MS in positive ion mode. Figure S2: The total ion chromatograms (TIC) of representative seaweed samples (QC) based on UPLC-Q-TOF/MS in negative ion mode. Table S1. Standard curves, linear ranges, Recovery, LODs and LOQs of arsenic (As). Table S2. Major biomarkers identified in two different seaweeds by liquid chromatography quadrupole time-of-flight mass spectrometry (LC-QTOF-MS).
