# Supplementary material for: Mass Spectrometry-Based Metabolomics Investigation on Two Different Seaweeds Under Arsenic Exposure
Source: Foods. 2024 Dec 16;13(24):4055. doi: 10.3390/foods13244055 (PMC11675553; doi:10.3390/foods13244055)
Supplement: Supplementary file 1 [file foods-13-04055-s001.zip › Table 1S.pdf]

Table 1S Standard curves, linear ranges, Recovery, LODs and LOQs of arsenic (As).

Element	Arsenic (As)
Standard curves	Y=0.9107X-0.9129
Linear ranges	20~4000
r	0.9992
Recovery	97.2%
Precision	0.96%
LOD	0.005
LOQ	0.017
