# Supplementary material for: Mass Spectrometry-Based Metabolomics Investigation on Two Different Seaweeds Under Arsenic Exposure
Source: Foods. 2024 Dec 16;13(24):4055. doi: 10.3390/foods13244055 (PMC11675553; doi:10.3390/foods13244055)
Supplement: Supplementary file 1 [file foods-13-04055-s001.zip › Table 2S.pdf]

Table 2 Major biomarkers identified in two different seaweeds by liquid chromatography quadrupole time-of-flight mass spectrometry (LC-QTOF-MS)

No.	Metabolites	Molecular formula	RT (min)	Ionization (ESI+/ESI−)	Observed (m/z)	Theoretical (m/z)	Error (ppm)	MS/MS fragments
1	Byrsonic acid	C26 H48 O6	10.637	[M+NH4]+	474.37951	474.378915	1.2542	
2	Glyceryl linolenate	C21 H36 O4	10.721	[M+H]+	353.26903	353.268636	1.1146	261.22127,243.21077
3	Methylpyrrolidone	C5 H9 N O	10.874	[M+H]+	100.0756	100.07569	-0.9033	58.02859,100.07552，
4	LDGTS (18:1)	C28 H53 N O6	10.895	[M+H]+	500.39455	500.394565	-0.0302	500.39442,236.14953
5	Stearyldiethanolamine	C22 H47 N O2	11.021	[M+H]+	358.36788	358.367956	-0.2131	
6	Lysyl-seryl-valine	C14 H28 N4 O5	11.070	[M+H]+	333.21298	333.213247	-0.8001	
7	LDGTS 20:2	C30 H55 N O6	11.092	[M+H]+	526.41015	526.410215	-0.1238	
8	N-Ethylcyclohexylamine	C8 H17 N	11.100	[M+H]+	128.14323	128.143376	-1.1398	128.14364,83.08612
9	3-O-alpha-L-rhamnopyranosyl-3-hydroxynonanoyl-3-hydroxydecanoic acid	C25 H46 O9	11.119	[M+NH4]+	508.34816	508.348009	0.2974	311.25733,237.22126
10	Glyceryl arachidonate	C23 H38 O4	11.589	[M+H]+	379.28413	379.284286	-0.4121	287.23681,269.22632
11	Linolenic Acid	C18 H30 O2	15.037	[M+H]+	279.23256	279.231857	2.5185	95.08533,81.06959,109.10083,123.11661,261.22216
12	Arachidonic Acid	C20 H32 O2	16.839	[M+H]+	305.24829	305.247507	2.5657	91.05407,105.06969,121.10104,305.24699
13	Betaine	C5H11NO2	0.686	[M+H]+	118.08629	118.086255	0.2955	58.06476,59.07257
14	Mannitol	C6H14O6	0.802	[M+K]+	221.04216	221.042197	-0.1678	102.94331,143.96858
15	Adenosine	C10H13N5O4	0.934	[M+H]+	268.10399	268.10403	-0.1508	136.06155,119.03509
16	Tyramine	C8 H11 N O	0.986	[M+H]+	138.09126	138.09134	-0.5827	77.03833,91.05404
17	Tyrosine	C9 H11 N O3	0.987	[M+H]+	182.08102	182.08117	-0.8223	91.05397,95.04897,119.04913,136.07540,123.04385

18	l-Isoleucine	C6 H13 N O2	1.028	[M+H]+	132.10195	132.101905	0.3393	86.09618,69.06968
**No.**	**Metabolites**	**Molecular formula**	**RT (min)**	**Ionization (ESI+/ESI−)**	**Observed (m/z)**	**Theoretical (m/z)**	**Error (ppm)**	**MS/MS fragments**
19	Phenylalanine	C9H11NO2	1.734	[M+H]+	166.0863	166.086255	0.2703	103.05396,120.08056
20	Adenine	C5H5N5	2.302	[M+H]+	136.06167	136.061772	-0.747	119.03513,136.06136
21	Tryptophan	C11 H12 N2 O2	2.450	[M+H]+	205.09706	205.097154	-0.459	118.06484,146.05973
22	Loliolide	C11H16O3	4.595	[M+H]+	197.11725	197.117221	0.1476	91.05401,105.06942
23	Cyclic pentaleucine	C30 H55 N5 O5	4.778	[M+H]+	566.4279	566.427597	0.5357	566.42734
24	Cyclic hexaleucine	C36 H66 N6 O6	5.158	[M+H]+	679.51174	679.511661	0.1169	679.51149
25	Cycloheptaleucine	C42 H77 N7 O7	5.429	[M+H]+	792.59552	792.595725	-0.2581	792.59537
26	Cyclo(L-isoleucyl-L-isoleucyl-L-isoleucyl-L-isoleucyl)	C24 H44 N4 O4	5.564	[M+H]+	453.34329	453.343533	-0.535	453.34333
27	Atractylenolide I	C15H18O2	7.861	[M+H]+	231.13807	231.137956	0.4917	175.07607,129.06952,141.07043,128.06176,115.05416,91.05402
28	artemisinin	C15 H22 O5	8.285	[M+H]+	283.15381	283.154	-0.6723	149.05985,107.08537,125.09577,181.12203
29	Atractylenolide III	C15 H20 O3	9.316	[M+H]+	249.14853	249.148521	0.0359	91.05389,93.06933,79.05333,105.07014,77.03739
30	Tetrahydrodeoxycortisol	C21 H34 O4	9.320	[M+H]+	351.25299	351.252986	0.0109	105.06959,119.08547
31	Lauroyl lysine	C18 H36 N2 O3	9.483	[M+Na]+	351.26179	351.261815	-0.0705	105.06966,119.08535
32	Butanedioic acid	C28 H49 N O6	9.748	[M+H]+	496.36374	496.363265	0.9571	496.36365,236.14913
33	Ganoderenic acid E	C30 H40 O8	10.225	[M+Na]+	551.26133	551.26154	-0.3811	551.26004,381.19212
34	Ascorbyl oleate	C24 H40 O7	10.321	[M+H]+	441.2847	441.28468	0.0447	259.20607,277.21646,
35	L-Ascorbyl stearate	C24 H42 O7	10.998	[M+H]+	443.30013	443.30033	-0.4519	261.22093,129.05441,279.23305,407.28049
36	Timnodonic acid	C20 H30 O2	11.614	[M+H]+	303.23204	303.231857	0.6043	91.05395,105.06979

No.	Metabolites	Molecular formula	RT (min)	Ionization (ESI+/ESI−)	Observed (m/z)	Theoretical (m/z)	Error (ppm)	MS/MS fragments
37	Diisobutyl phthalate	C16H22O4	12.021	[M+H]+	279.15907	279.159086	-0.0564	149.02318, 223.05634
38	Pentaerythritol tetra(2-ethylhexanoate)	C37 H68 O8	13.392	[M+NH4]+	658.52516	658.525245	-0.129	658.52512
39	Stearidonic acid	C18 H28 O2	13.488	[M+H]+	277.2155	277.216207	-2.5493	91.05397,93.06966,105.06961
40	Dysolenticin C	C27 H38 O5	13.854	[M+Na]+	465.26126	465.261146	0.2448	311.25807,155.01016,447.25111
41	Petrosaspongiolide m	C27 H40 O6	14.018	[M+H]+	461.28959	461.289766	-0.3807	267.21065,285.22118,461.28845
42	Linoleamide	C18 H33 N O	14.063	[M+H]+	280.26358	280.263491	0.3167	95.08522,109.10106,119.08487
43	2-Arachidonoylglycerol	C23 H38 O4	14.830	[M+H]+	379.28458	379.284286	0.7743	269.22644,91.05384,287.23688
44	Retinol	C20 H30 O	14.877	[M+H]+	287.23694	287.236942	-0.0074	91.05348,105.06942,145.10007
45	hyousterone D	C27 H42 O6	15.288	[M+H]+	463.30537	463.305416	-0.0986	287.23692,269.22598,203.17919
46	hyousterone C	C27 H42 O6	15.805	[M+H]+	463.30582	463.305416	0.8726	287.23691,269.22606,203.17941
47	Fuscoside B	C25 H40 O5	16.288	[M+H]+	421.29499	421.294851	0.3299	141.05463,85.02838,421.09480
48	Oleamide	C18 H35 N O	16.583	[M+H]+	282.28064	282.279141	5.3092	97.10092,107.08533,149.13232,
49	Pheophorbide a	C35 H36 N4 O5	17.978	[M+H]+	593.27604	593.275847	0.3255	593.27616,533.25494,460.22583
50	Dihomo-gamma-linolenic acid	C20 H34 O2	18.413	[M+H]+	307.26327	307.263157	0.3681	95.08531,109.10107,123.11627, 271.24400,307.25936,289.25153
51	Stearyl gallate	C25 H42 O5	18.842	[M+H]+	423.3104	423.310501	-0.2388	141.05401,85.02833,265.25271
52	4-(Hydroxymethyl)benzyl-3beta -cholestanyl succinate	C39 H60 O5	19.016	[M+H]+	609.45094	609.451352	-0.6755	333.24568,273.25621,591.44164
53	(1S,2R,4aR,6aR,6bS,12aS,14bR)-1,2,4a,6a,6b,9,12a-Heptameth yl-1,2,3,4,4a,5,6,6a,6b,7,8,8a,11 ,12,12a,14b-hexadecahydropice ne	C29 H44	19.572	[M+H]+	393.35177	393.351578	0.4881	393.35157,147.11632,151.13252

No.	Metabolites	Molecular formula	RT (min)	Ionization (ESI+/ESI−)	Observed (m/z)	Theoretical (m/z)	Error (ppm)	MS/MS fragments
54	Ergocalciferol	C28H44O	19.718	[M+H]+	397.34612	397.346493	-0.9378	379.33967,397.34358,109.98240
55	(3α,12β,24R)-12-Acetoxy-25-hydroxy-20,24-epoxydammaran-3-yl3-(nonylamino)-3-oxopropanoate	C44 H75 N O7	20.032	[M+H]+	730.56189	730.56163	0.3552	502.35185,144.10164,100.11256
56	Oleic acid	C18 H34 O2	20.077	[M+H]+	283.26319	283.263157	0.1168	55.67505,67.05492,77.03826
57	28-Noroleana-12,17-dien-3-one	C29 H44 O	20.338	[M+H]+	409.34662	409.346493	0.3112	283.24289,409.34627,309.25770
58	Sucrose	C12 H22 O11	29.560	[M+Na]+	365.1054	365.105433	-0.0913	203.05283,365.10586,213.82599
59	Fucoxanthin	C42 H58 O6	15.764	[M+H]+	659.42868	659.430616	-2.9363	109.10128,659.42578,641.41790
60	DL-Proline	C5 H9 N O2	1.386	[M+H]+	116.07057	116.070605	-0.3018	70.0654
61	Indole-3-carboxaldehyde	C9 H7 N O	2.497	[M+H]+	146.05989	146.060040	-1.0292	146.06006, 118.06468
62	Germacrone	C15 H22 O	0.576	[M+H]+	219.17446	219.174342	0.539	203.14446,219.17333
63	Violaceoid A	C14 H20 O3	6.78	[M+H]+	237.14856	237.148521	0.1643	91.05392,109.06484,159.11628
64	Riboflavin	C17 H20 N4 O6	3.458	[M+H]+	377.14502	377.145561	-1.4343	243.08748,377.14403,172.08661
65	linoleic acid	C18 H32 O2	12.070	[M-H]-	279.23203	279.232954	-3.3088	279.23227
66	palmitic acid	C16 H32 O2	13.202	[M-H]-	255.23226	255.232954	-2.7188	255.23119
67	gamma-linolenic acid	C18 H30 O2	10.983	[M-H]-	277.21641	277.217304	-3.2244	277.21586
68	L-Pyroglutamic acid	C5 H7 N O3	0.929	[M-H]-	128.03465	128.035317	-5.207	128.03521
69	3-Furoic acid	C5 H4 O3	0.919	[M-H]-	111.00798	111.008768	-7.0947	67.01810
70	Ilekudinol B	C29 H44 O4	21.797	[M-H]-	455.31582	455.316684	-1.8967	411.32581,455.31343
71	3,4-Dihydroxybenzoic acid	C7 H6 O4	2.100	[M-H]-	153.01874	153.019332	-3.8706	108.02150,109.02882
72	Benzoic Acid	C7 H6 O2	3.643	[M-H]-	121.02886	121.029503	-5.3129	121.02888,93.03483
73	2-Hydroxyphytanic acid	C20 H40 O3	19.228	[M-H]-	327.28915	327.290469	-4.0296	185.00772,327.18418
