# Supplementary figures and images for: Mass Spectrometry-Based Metabolomics Investigation on Two Different Seaweeds Under Arsenic Exposure
Source: Foods. 2024 Dec 16;13(24):4055. doi: 10.3390/foods13244055 (PMC11675553; doi:10.3390/foods13244055)

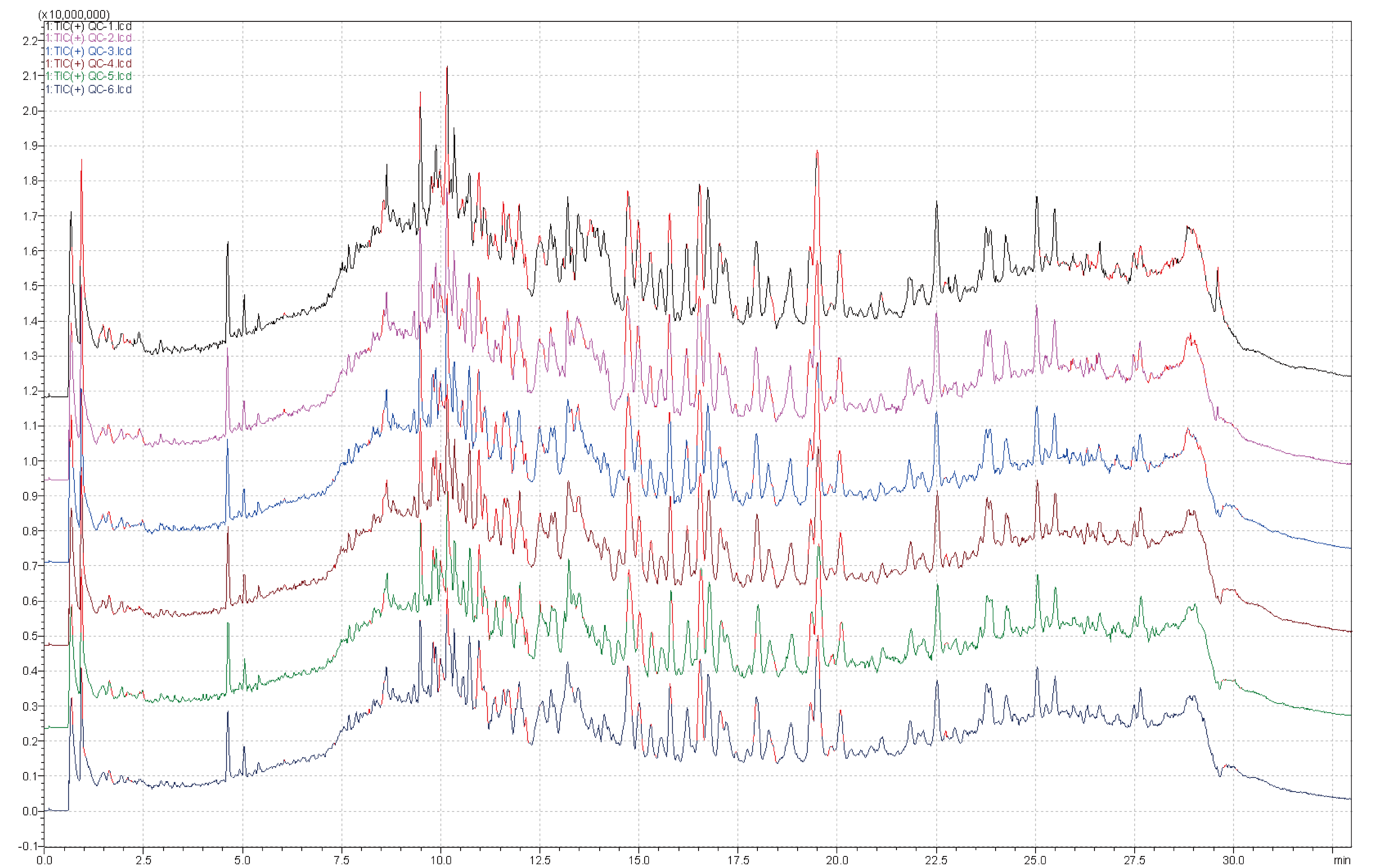

Supplement: Supplementary file 1 [file foods-13-04055-s001.zip › Figure 1S.png]

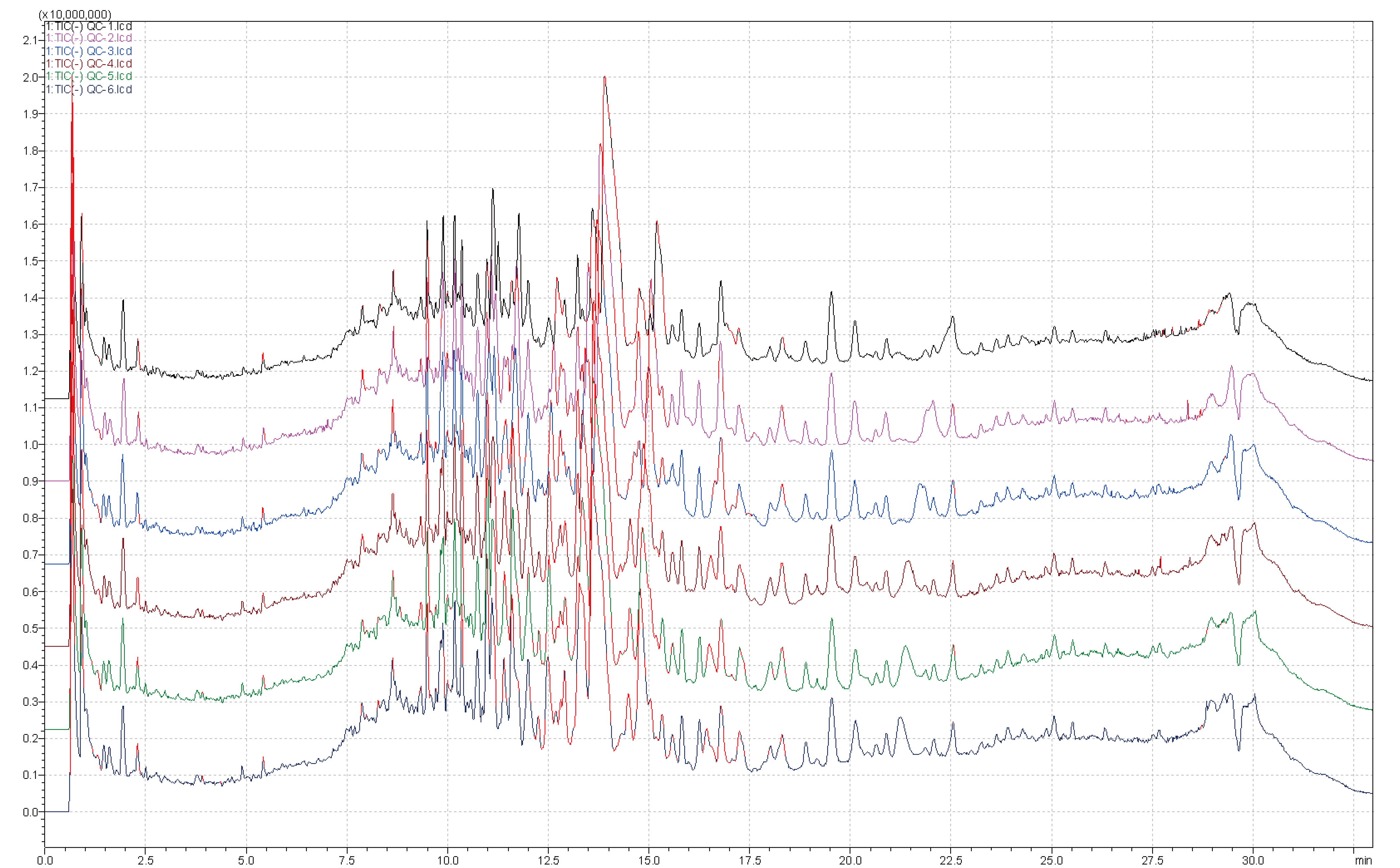

Supplement: Supplementary file 1 [file foods-13-04055-s001.zip › Figure 2S.png]
